# First autochthonous case of *Opisthorchis felineus* in Austria

**DOI:** 10.1186/s13071-025-06659-5

**Published:** 2025-01-21

**Authors:** Lisa-Maria Kulmer, Maria Sophia Unterköfler, Yasamin Vali, Ilse Schwendenwein, Nicole Luckschander-Zeller

**Affiliations:** 1 Department for Companion Animals and Horses, University Hospital for Small Animals, University of Veterinary Medicine Vienna, Vienna, Austria; 2 Department of Biomedical Sciences and Pathobiology, Institute of Parasitology, University of Veterinary Medicine Vienna, Vienna, Austria; 3 Department for Companion Animals and Horses, Diagnostic Imaging, University of Veterinary Medicine Vienna, Vienna, Austria; 4 Clinical Pathology Platform, University of Veterinary Medicine, 1210 Vienna, Austria

**Keywords:** Liver fluke infestation, Opisthorchiasis, *Opisthorchis felineus*, Zoonosis, Feline liver fluke, Cholangitis, Austria

## Abstract

**Background:**

*Opisthorchis felineus* is a feline pathogen with zoonotic potential that can be a causative agent of human opisthorchiasis and cholangiocarcinoma. In Europe, *O. felineus* is particularly endemic in Eastern European countries, while this parasite has also been sporadically detected in Germany, Italy and northern Poland. Parts of Asia, such as Russia, Ukraine and Kazakhstan, are also affected.

**Methods:**

A 7-year-old female neutered European Shorthair cat, without any traveling history, presented in May 2023 with weight loss, anorexia and vomiting.

**Results:**

The cat showed increased liver enzyme activities, hyperbilirubinemia and hyperammonemia consistent with the suspected diagnosis of cholangitis with consecutive hepatoencephalopathy. Eggs of *O.* *felineus* were detected by routine cytological examination of bile smears and PCR confirmed *O. felineus*.

**Conclusions:**

This is the first report of autochthonous *O. felineus* infection in Austria.

**Graphical Abstract:**

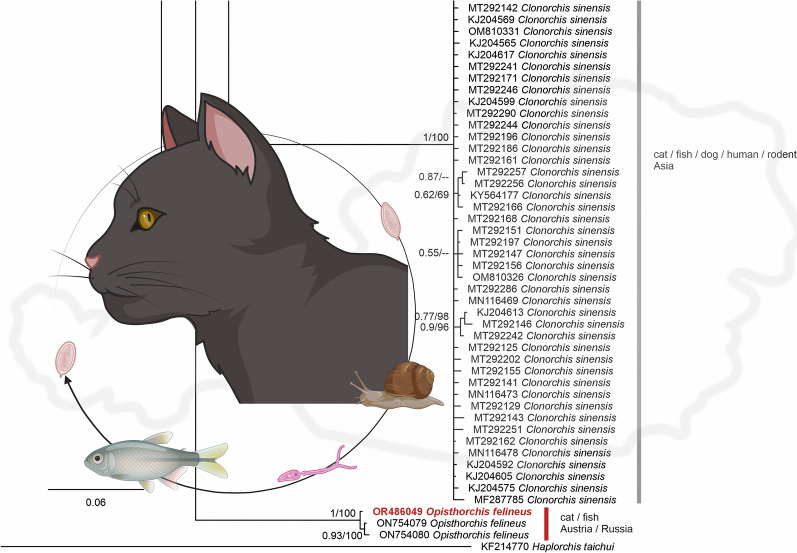

## Background

*Opisthorchis felineus*, belonging to the family of Opistorchiidae, are small liver flukes that can infect humans, causing human opisthorchiasis. Human liver fluke infections are endemic in Eurasia, and the WHO estimated that 1.51 million people are currently infected with *O.* *felineus* in that region [1]*.* In Southeast Asia, other members of the family Opisthorchiidae can be found in up to 50% of free-roaming cats. Reports of clinical disease in cats are uncommon, but may be underreported. In Europe, *O. felineus* has been reported in several countries, but to our knowledge there have as yet been no reports of cats infected with *O. felineus* in Austria [2–5]. In Siberia, this liver fluke is an important zoonotic pathogen, with a reported prevalence exceeding 80% in humans in some regions. A study from Western Siberia reported that people infected with *O. felineus* have a significantly increased risk of developing cholangiocarcinoma [6]. However, reports of this specific liver fluke are rare in cats, and the life-cycle is likely maintained by wild carnivores, as it has been found in various species, including the abundant red fox (*Vulpes vulpes*) [7].

Mammals serve as definitive hosts for this parasite. Following infection, the fluke migrates to the liver of the infected host, and eggs are shed with the bile into feces [7]. The fluke requires two intermediate hosts: freshwater snails (*Bithynia* spp.), which are the first intermediate host, and cyprinid fishes, which are the second intermediate host. The consumption of raw fish is the main route of infection for new definitive hosts [8].

Most infected cats are asymptomatic, and clinical symptoms, if present, are highly variable and non-specific. Specific symptoms include jaundice, dehydration, hepatomegaly, ascites, inappetence, vomiting, diarrhea and weight loss, among others. These clinical symptoms resemble those of lymphocytic cholangitis. Parasite burden, the time of infection and the animal’s response to parasite infection interact in complex ways, influencing the overall health and clinical presentation of the infected cat [8]. Chronic infections or high parasite burden causing hepatic failure and extrahepatic biliary duct obstruction can result in death of the infected animal [9]. Laboratory test results which can be indicative of infection include mild non-regenerative anemia, eosinophilia, lymphocytosis, hyperbilirubinemia and increased alkaline phosphatase (ALP) and alkaline transaminase (ALP) activities [4]. Abdominal radiographs and ultrasonograms are often unremarkable and/or non-specific. Findings may include distended bile ducts, hyperechoic and thickened gallbladder wall, gallbladder “sludge”, hepatomegaly and/or diffuse cystic liver changes [10]. Praziquantel is the anthelmintic of choice for treating *O. felineus*, with dosages ranging from 20 to 40 mg/kg injected subcutaneously or intramuscularly or administered orally as a single dose or every 24 h for 3–5 days [9, 11]. In cats infected with feline immunodeficiency virus a concurrent bacterial cholangitis with *Escherichia coli* has been reported [10]. Liver fluke infestation is rarely associated with biliary cystadenoma or cholangiocarcinoma in cats [12, 13].

Diagnosis is made by fecal flotation or sedimentation analysis, but the results can be compromised by intermittent egg shedding, low parasite burdens and variable fecal analysis methods [14]. Diagnostic sensitivity can be increased by analysis of bile obtained by percutaneous US-guided cholecystocentesis [10]. A very small sample of bile (10–20 µL) in a direct smear preparation is considered sufficient to identify eggs [15].

## Methods

### Cytology

Percutaneous US-guided cholecystocentesis and fine-needle aspiration of the liver were performed. Routine hematoxylin-eosin staining and bacteriological culture of liver and bile samples were also performed.

### Molecular analysis

Bile samples were examined under a light microscope, and DNA was extracted using a commercial DNA extraction kit (QIAamp PowerFecal Pro DNA Kit; QIAGEN, Hilden, Germany) according to the manufacturer’s instructions, with the adaption that the TissueLyser II system (QIAGEN) was used for mechanical homogenization. To obtain a fragment of the mitochondrial cytochrome* c* oxidase subunit I (COI) gene consisting of 638 nucleotides, we performed PCR tests using the primers COINeodFw (5′-TTT ACT TTG GAT CAT AAG CG-3′) and COINeodRv (5′-CCA AAA AAC CAA AAC ATA TGT TGA A-3′) [16], using the following cycling profile: an initial denaturation at 95 °C for 2 min; followed by 35 cycles of 95 °C, 48 °C and 72 °C, each for 1 min; and a final extension at 72 °C for 5 min. The PCR product was run on 2% agarose gels stained with Midori Green, following which it was sent to Microsynth Austria GmbH (Vienna, Austria) for purification and sequencing in both directions. The electropherograms were inspected by eye using BioEdit [17]. The sequence was compared to available sequences using the GenBank nucleotide basic local alignment search tool (BLAST). For the phylogenetic analysis, nucleotide sequences of members of family Opisthorchiidae (taxid: 6196) available on the National Center for Biotechnology Information (NCBI) GenBank database were searched with the BLASTn function, using the sequence obtained in this study. The sequences were aligned and cut to the same length using BioEdit [17]. Maximum likelihood (ML) and Bayesian inference (BI) trees were calculated based on the alignment, including 310 sequences (507 nucleotide positions). Sequences were collapsed to haplotypes using DAMBE v.7.0.5.1 [18], leaving 83 haplotypes. A sequence of *Haplorchis taichui* (GenBank accession number: KF214770) was used as outgroup. A ML bootstrap consensus tree (1000 replicates) was calculated using the W-IQTREE web server (http://iqtree.cibiv.univie.ac.at/; [19]) applying the model K3Pu + F + I + G4, which was suggested as the best fit for the data set in the model test according to the corrected Akaike information criterion. The BI trees were calculated using MrBayes v.3.2.7 [20], applying the next complex model GTR + I + G, because the same model was not available in this program. The analysis was run for 10^6^ generations (number of chains: 4), sampling every 1000th tree. The first 25% of trees were discarded as burn-in, and a 50% majority-rule consensus tree was calculated based on the remaining 7500 trees.

## Results

### Clinical presentation

A 7-year-old female neutered European Shorthair cat presented to the emergency service for small animals at Vetmeduni Vienna in May 2023 because of severe weight loss, vomiting and ongoing inappetence. The cat had been owned by its carer since it was a kitten and was from a farm in Lower Austria. It cat was neither regularly vaccinated nor dewormed, had no history of periods abroad and was treated as an indoor-only cat. The cat had been fed conventional cat food and had never had access to raw fish or fish scraps. The other two cats in the household appeared to be clinically normal. Preliminary treatment by the referring veterinarian included cefovecin, meloxicam and mirtazapine. Clinical examination of the cat revealed mildly icteric mucous membranes and moderately reduced skin elasticity.

### Blood tests

Blood tests were performed following presentation, revealing an unremarkable complete blood count. Bilirubin concentration and liver enzyme activities, including ALT and ALP, were increased. To test liver function, whole blood ammonia concentration was measured and found to be significantly increased (Table 1). Serum amyloid A level was within the reference range at 2 mg/L (reference range < 5.0 mg/L according to laboratory's reference intervals, Vetmeduni Vienna). Prior to further diagnostic assessment a coagulation status was performed; the prothrombin time was found to be significantly prolonged to 14.5 s (reference range 8.0–10 0.0 s, according to laboratory's reference intervals, Vetmeduni Vienna). Partial thromboplastin time (PTT) as well es the thrombin time (TZ) were within their respective reference range**.** Results of the Combo + Snaptest® (Idexx Laboratories, Westbrook, ME, USA) for infection with feline immunodeficiency virus (FIV) and feline leukemia virus (FeLV) were negative.

**Table 1:** Blood chemistry results—May and June 2023

Parameter	May 2023	June 2023	From	To
ALP (U/L)	464	48	–	< 30.00
ALT (U/L)	293	56	–	< 100.00
Bilirubin (mg/dL)	7.41	0.14	–	< 0.2
Total protein (g/dL)	7.21	7.69	6.00	7.50
Albumin g/dL	3.28	3.61	2.60	4.60
Ammonia (µmol/L)	129	28	–	< 60.00

*ALP* Alkaline phosphatase,* ALT* alanine aminotransferase

### Abdominal ultrasonography and ultrasonography-guided fine-needle aspiration

**Fig. 1:**
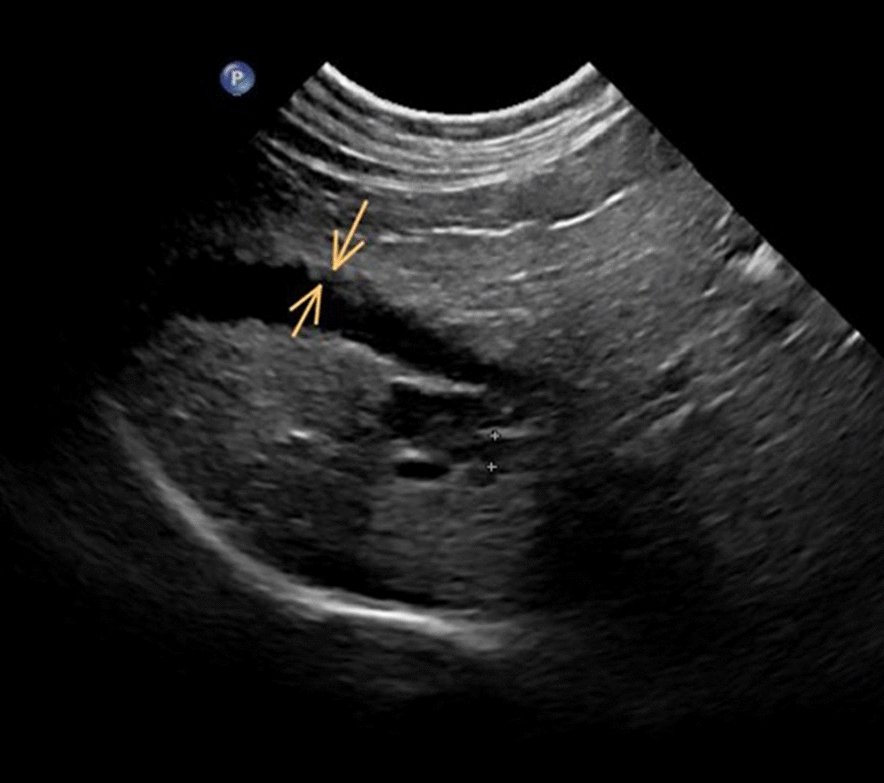
Ultrasonogram showing an irregular thickening of the gall bladder wall (space between the arrows) and mild to moderate distension of the common bile duct (between the cursors [*+*])

**Fig. 2:**
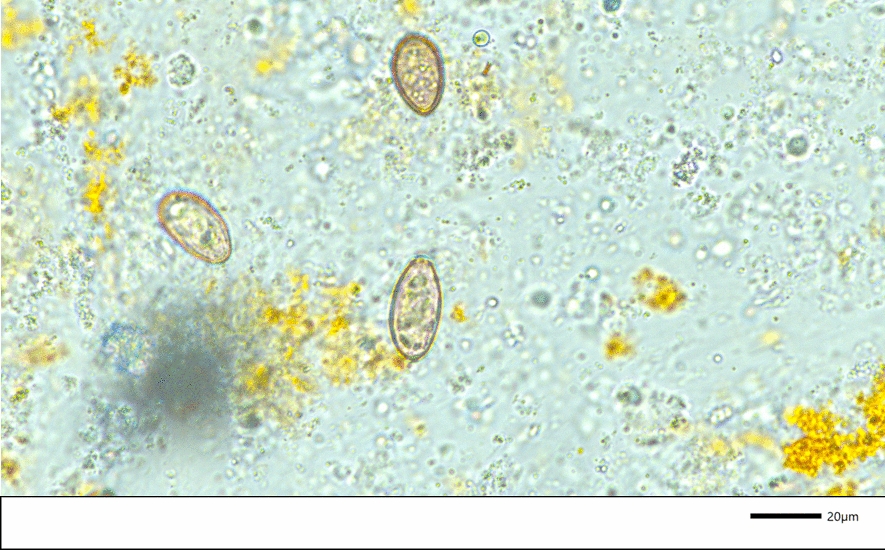
*Opisthorchis felineus* egg (magnification 600×) in an unstained wet mount of bile fluid

To provide additional information for the diagnostic work-up, a European board-certified radiologist performed an abdominal US. The ultrasonogram showed that the liver was significantly enlarged with homogenous parenchyma. The gall bladder wall had an irregular lining, and the common bile duct (CBD) showed a width of 2.2 mm (< 4.0 mm) without any signs of obstruction (Fig. 1) Based on these findings consent from the owner was sought to perform an US-guided-fine-needle aspiration of the liver and cholecystocentesis for cytologic and bacteriologic examination. Liver cytology revealed numerous clusters of swollen hepatocytes with signs of fatty degeneration; also, neutrophilic infiltrates were seen in some areas of the liver. Examination of bile fluid smears revealed operculated, colorless parasitic objects measuring 26.8–31.0 × 13.4–14.6 µm, which is consistent with the size of *O.* *felineus* eggs found (Fig. 2).

#### PCR and phylogenetic tree

**Fig. 3:**
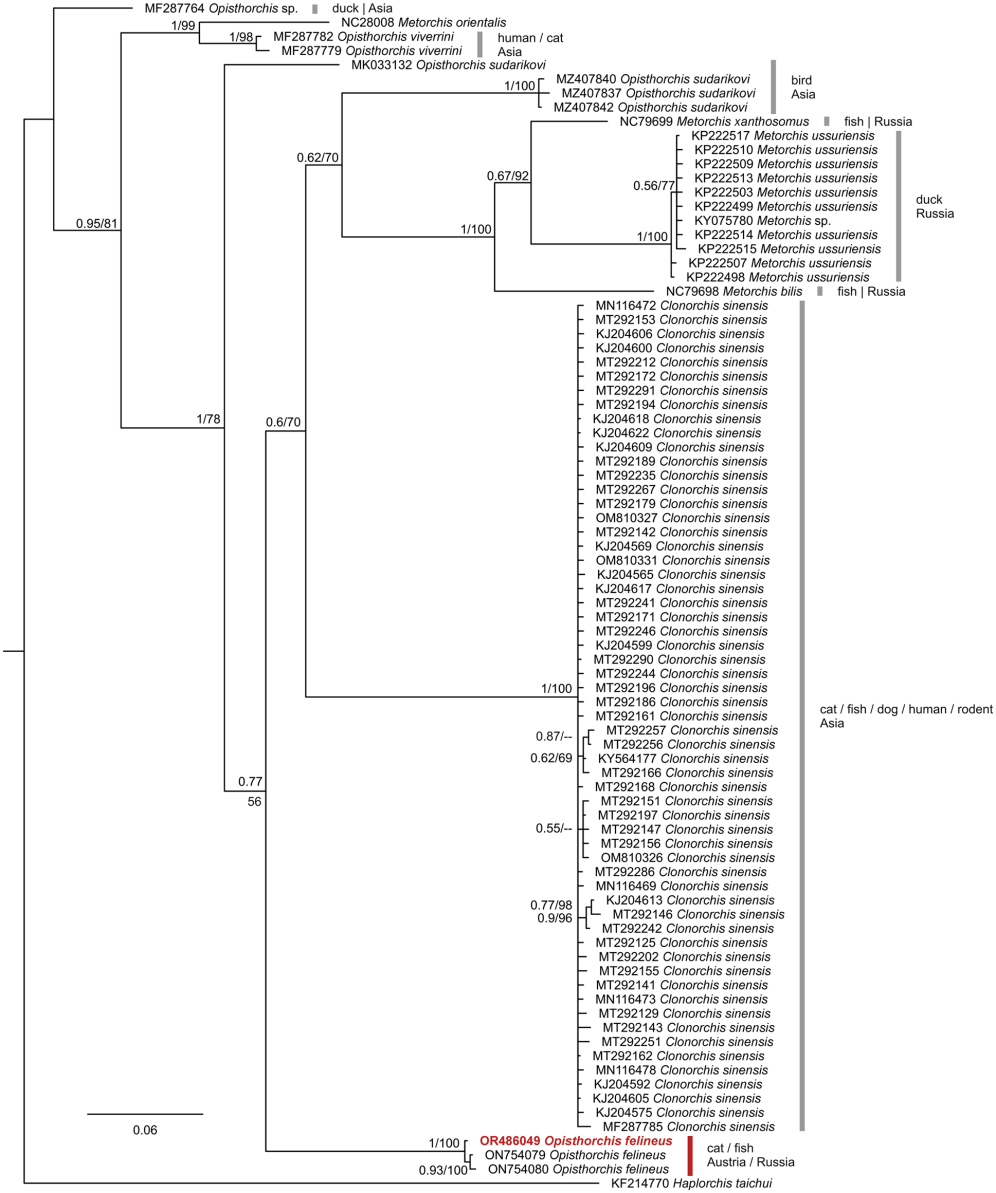
Bayesian interference (BI) tree featuring mitochondrial cytochrome* c* oxidase subunit I sequences (507 nucleotides) of members of family Opisthorchiidae. BI posterior probabilities and maximum likelihood (ML) bootstrap values are indicated at each node (BI posterior probability/ML). The sequence marked in red was obtained in the present study. For each sequence, the GenBank accession number, species name, host and country of origin are provided. The scale bar indicates the expected mean number of substitutions per site according to the model of sequence evolution applied

A PCR test was performed to confirm further that *O. felineus* was involved in the clinical presentation. The DNA sequence obtained was 100% identical to an *O. felineus* sequence documented in a cat from Russia (GenBank accession number: NC011127) and has been uploaded to BoldSystems® web platform (Process ID: PAVEA251-23) and GenBank (accession number: OR486049). In the phylogentic tree the sequence was within a clade with other *O. felineus* found in fishes (Fig. 3).

### Treatment and outcome

The cat was treated with praziquantel 5 mg/kg every 24 h, by constant rate infusion dosing, for 3 days. As symptomatic therapy, the cat was also treated with 1 mg/kg maropitant every 24 h, ondansetron 0.2 mg/kg every 8 h, S-adenosin-methionin 20 mg/kg every 24 h, ursodeoxycholic acid 10 mg/kg every 24 h, marbofloxacin 2 mg/kg every 24 h and lactulose 150 mg/cat every 8 h. After the first dose of praziquantel, the cat developed anisocoria with absent menace response and pupillary reflex, as well as generalized tremor and a head tilt consistent with a hepatoencephalic syndrome. To prevent the progression of hepatic lipidosis, the cat was fed via nasogastric tube. The neurological symptoms were self-limiting, and a significant improvement was observed after 3 days of therapy. The cat started eating on its own and was discharged home after a total of 8 days. At the follow-up examination after 1 month, the cat had already gained 1 kg bodyweight, and the clinical examination was unremarkable. Laboratory test results showed improvements, and the ammonia concentration was within the reference limit. The pooled stool sample collected over 3 days was negative for *O. felineus*.

## Discussion

To our knowledge this is the first case report of an autochthonous infection with *O. felineus* in a cat in Austria. Cats are considered to be important reservoir hosts for human opisthorchiasis, particularly in areas with a high prevalence of infection [5]. While travel history and/or importation from endemic regions may be important risk factors in dogs, these factors may play a minor role in cats. Also, our patient has no history of time outside the country.

The clinical symptoms in cats usually correlate with the worm burden. If a cat has a high parasite load, it will exhibit symptoms such as lethargy, diarrhea, ocular and nasal discharges. Age, sex and breed are not related to the degree of infection [2]. Cats in endemic areas are usually raised without annual health checks and deworming, and the prevalence of members of family Opisthorchiidae in such areas is 30–50% in free-roaming cats, with cats possibly acting as reservoir host [2, 4]. One possible source of infection for cats is the consumption of self-caught fish from natural water reservoirs or fish scraps from village households [11].

In our case, the cat was adopted from a farm in Lower Austria and reportedly has never eaten raw fish or fish scraps. Additionally, there was no large water source in the surrounding area of the farm. Therefore, the source of the liver fluke infestation remains unclear. However, it is possible that the cat became infected as a kitten, as it was kept indoors thereafter. Metacercaria has been reported to be able to remain in fish muscle and subcutaneous tissue for up to 7 years or more [21]; adult liver flukes in humans can survive for 7–15 years [22].

Even though the cat owners stated that they had not fed raw or frozen fish to the cat and had never engaged in fishing, either as a profession or as a hobby, the consumption of fresh or frozen fish by the cat cannot be completely ruled out, with the cat becoming infected at a later time point. Another, rather unlikely possibility could be an unnoticed inadequate processing of the commercial feed. However, these are speculations only; the exact route of infection in this cat remains unclear. Further studies in the prevalence of *O. felineus* in Austria and studies into the longevity of adult trematodes are necessary to be able to estimate the probability of the source of infection.

Thus, the origin of infection of the patient remains unclear. The predominant endemic area for *O. felineus* is Russia, with approximately 1.22 million people infected in the 1990s [1]. A huge outbreak of human opisthorchiasis was reported after ingestion of raw fish from lake Bolsena and Bracciano in Italy [23]. In humans, infections with *O. felineus* are occasionally reported in other parts of the world due to human immigration from endemic countries, consumption of raw or undercooked freshwater fish or the import of the freshwater fish from endemic areas [1].

Our cat exhibited an acute disease course, which could have been triggered by the concurrent hepatic encephalopathy (lethargy, inappetence) and superimposed hepatic lipidosis. Ultrasonography revealed a mildly distended CBD without signs of obstruction. Thickening of the gallbladder wall and distension of the common bile duct without obstruction are ultrasonographic findings associated with cholecystitis and cholangitis, regardless of etiology. Based on published ultrasonographic parameters, the upper limit for CBD diameter in normal and icteric cats is up to 4 mm. A CBD diameter > 5.0 mm is suggestive of extrahepatic bile duct obstruction [24, 25]. However, thickening of the gallbladder wall and distension of the CBD are pathognomonic for parasitic infection, as shown in a case series published by Haney et al. [26] in 2006. These authors found that all cats with liver fluke infections had a significantly enlarged CBD as evidenced on the ultrasonogram and by pathohistology, which exhibited the consequent inflammation.

The diagnosis of a feline liver fluke infestation is typically made through examination of a pooled stool sample. In our case, the parasite eggs were found in the bile. Köster et al. [10] evaluated percutaneous ultrasonography-guided cholecystocentesis and bile analysis for the detection of *Platynosomum* spp., and found out that bile egg counts were more sensitive than fecal egg counts for diagnosing platynosomosis. In our study, diagnosis was made on the second day of the cat's hospital stay and there was also not enough stool material available at that time for a significant stool examination. Consequently, diagnosing the infection based on examination of the bile was significantly faster, although associated with higher risk of bleeding and leakage of bile into the abdomen [27].

The anthelmintic drug of choice for treating *O. felineus* is praziquantel with a dosage range of 20–40 mg/kg, given as a single dose or every 24 h for 3–5 days. Praziquantel is considered to be a safe drug [9, 11], and Sereerak et al. [11] reported that in their study they did not observe any side effects in healthy cats given a single dose of 40 mg/kg. Our feline patient developed significant neurological symptoms, including blindness and generalized tremors, after administration of the first dose of 5 mg/kg every 24 h for 3 days. Since praziquantel is primarily metabolized by the liver, its administration could have temporarily exacerbated hepatic encephalopathy, leading to the pronounced neurological symptoms of the cat. Although studies have shown that a single dose of praziquantel at 30 mg/kg in dogs with clonorchiasis only achieved a cure rate of 20%, our patient's pooled stool sample was negative for infection after 1 month [28]. A repeat percutaneous ultrasound-guided cholecystocentesis was deemed unnecessary at follow-up since the cat was clinically normal and had already gained 1 kg within the month. However, *O. felineus* is only shed intermittently in feces and there is always a possibility of a false negative stool sample. At the time of writing the case report, the cat continues to be doing well and appears to be clinically normal.

## Conclusions

This case report presents the first autochthonous infection of a cat with *O. felineus* in Austria. Veterinarians should consider infection with *O. felineus* in cats with the suspected diagnosis of cholangitis, despite Austria not yet considered to be an endemic area for *O. felineus*. This case report highlights that fine-needle aspiration of the gallbladder is essential as an advanced diagnostic procedure in cats with suspected cholangitis, as the diagnosis could not have been correctly made otherwise and the infection with *O. felineus* would have been missed. In cats diagnosed with *O. felineus* and associated hepatoencephalic syndrome, caution should be applied with the dosage of praziquantel, as in our patient its administration led to pronounced neurological symptoms. To determine the prevalence of *O. felineus* in Austria, follow-up studies involving cats, humans and fishes are necessary to raise awareness of this zoonotic parasite.

## Data Availability

No datasets were generated or analysed during the current study.

## Abbreviations

ALP: Alkaline phosphatase
ALT: Alanine transaminase
CBD: Common bile duct
FeLV: Feline leukemia virus
FIV: Feline immunodeficiency virus
PO: Per oral route
